# Hyperoxic Exposure Caused Lung Lipid Compositional Changes in Neonatal Mice

**DOI:** 10.3390/metabo10090340

**Published:** 2020-08-21

**Authors:** Abigail L. Peterson, Jennifer F. Carr, Xiangming Ji, Phyllis A. Dennery, Hongwei Yao

**Affiliations:** 1 Department of Molecular Biology, Cell Biology & Biochemistry, Division of Biology and Medicine, Brown University, Providence, RI 02912, USA; abigail_peterson@brown.edu (A.L.P.); jennifer_carr@brown.edu (J.F.C.); phyllis_dennery@brown.edu (P.A.D.); 2 Department of Nutrition, Byrdine F. Lewis School of Nursing and Health Professions, Georgia State University, Atlanta, GA 30302, USA; xji4@gsu.edu; 3 Department of Pediatrics, Warren Alpert Medical School of Brown University, Providence, RI 02912, USA

**Keywords:** bronchopulmonary dysplasia, lung alveolarization, lipidomics, metabolomics, oxidative stress

## Abstract

Treatments with supplemental oxygen in premature infants can impair lung development, leading to bronchopulmonary dysplasia (BPD). Although a stage-specific alteration of lung lipidome occurs during postnatal lung development, whether neonatal hyperoxia, a known mediator of BPD in rodent models, changes lipid profiles in mouse lungs is still to be elucidated. To answer this question, newborn mice were exposed to hyperoxia for 3 days and allowed to recover in normoxia until postnatal day (pnd) 7 and pnd14, time-points spanning the peak stage of alveologenesis. A total of 2263 lung lipid species were detected by liquid chromatography–mass spectrometry, covering 5 lipid categories and 18 lipid subclasses. The most commonly identified lipid species were glycerophospholipids, followed by sphingolipids and glycerolipids. In normoxic conditions, certain glycerophospholipid and glycerolipid species augmented at pnd14 compared to pnd7. At pnd7, hyperoxia generally increased glycerophospholipid, sphingolipid, and glycerolipid species. Hyperoxia increased NADPH, acetyl CoA, and citrate acid but reduced carnitine and acyl carnitine. Hyperoxia increased oxidized glutathione but reduced catalase. These changes were not apparent at pnd14. Hyperoxia reduced docosahexaenoic acid and arachidonic acid at pnd14 but not at pnd7. Altogether, the lung lipidome changes throughout alveolarization. Neonatal hyperoxia alters the lung lipidome, which may contribute to alveolar simplification and dysregulated vascular development.

## 1. Introduction

Approximately 1 in 10 babies are born prematurely each year in the US. Mechanical ventilation and supplemental oxygen are commonly employed to sustain life in these premature infants. Unfortunately, these therapies can impair lung development, which leads to continued dependency on supplemental oxygen beyond 36 weeks corrected gestational age, referred to as bronchopulmonary dysplasia (BPD) [1]. The pathology of BPD is characterized by alveolar simplification and dysregulated vascular development [1]. The mechanisms underlying alveolar simplification and dysregulated vascular development in BPD are not fully understood. Mouse lungs at birth are structurally similar to human neonates born at 30 to 34 weeks of gestation, when the lung is in the saccular phase of development. Thus, hyperoxic exposure in newborn rodents can be used to mimic lung injury in premature infants with BPD, and this model is frequently utilized to investigate pathogenesis and to identify potential therapeutic targets for hyperoxic lung injury in BPD [2,3,4].

Lipids are major structural components of biological membranes. Lipids also function as an energy reservoir and interact with proteins to modulate their functions. Furthermore, lipids are important molecules in modulating the immune system and mediating signal transduction. In the lungs, lipids are particularly important, as pulmonary surfactant contains 90% lipids and is essential for decreasing surface tension in the alveoli [5]. Pulmonary surfactant is commonly deficient in the immature lung of premature infants [6]. Early administration of surfactant reduces neonatal mortality and the occurrence of BPD [7]. Sphingolipid metabolites are increased in tracheal aspirates from preterm infants with BPD, which was associated with augmented apoptosis [8,9]. In addition, lipids can be attacked by reactive oxygen species, leading to lipid peroxidation, which plays important roles in the development of BPD [10]. Therefore, a thorough analysis using an untargeted omics investigation of lipid profiles is necessary to identify potential mechanisms for the pathogenesis of BPD.

A previous study has identified 924 unique lipids across 21 lipid subclasses in lungs from postnatal day (pnd) 7, pnd14, and adult mice, with dramatic alterations in the lipidome across developmental stages using lipidomics analysis [11]. For instance, a high content of monounsaturated lipid species is observed in lungs from mice at pnd1; myristic and palmitic acid-containing lipid species are present in lungs at pnd15, whereas adult lungs are enriched with polyunsaturated lipid species [12]. Although hyperoxic exposure decreases cellular content of monounsaturated and polyunsaturated fatty acids but increases levels of saturated fatty acids in cultured tracheal epithelial cells [13], there are no reports regarding whether neonatal hyperoxia changes lipid profiles in mouse lungs. In this study, we employed liquid chromatography–mass spectrometry (LC–MS) to profile lung lipid changes at pnd7 and pnd14 in mice exposed to hyperoxia as neonates. Furthermore, we complemented these untargeted lipidomics measurements with metabolomics analyses from the same samples to provide a more comprehensive picture of lipid metabolism after neonatal hyperoxic exposure. Finally, we measured lung glutathione and catalase to determine whether neonatal hyperoxia causes oxidative stress leading to lipid peroxidation at both pnd7 and pnd14.

## 2. Results

### 2.1. Overview and Analysis of the Pulmonary Lipidome in Response to Neonatal Hyperoxia

We used a lipidomics platform consisting of an Agilent 1100 series HPLC coupled with a Velos Orbitrap mass spectrometer operating in data-dependent MS/MS mode to profile the murine lung lipidome at pnd7 and pnd14 in mice exposed to hyperoxia as neonates (*n* = 6, each group). These time-points were chosen because they span peak stages of lung alveologenesis [14]. As shown in Table S1 and Table 1, 2263 lipid species were detected in lung homogenates, which covered 5 lipid categories and 18 lipid subclasses based on their MS/MS fragmentation patterns. The most commonly identified lipid species in the lung lipidome were glycerophospholipids, which included PA, PC, PE, PG, PI, PS, and CL. This was followed by sphingolipids and glycerolipids (Table 1).

**Figure 1:**
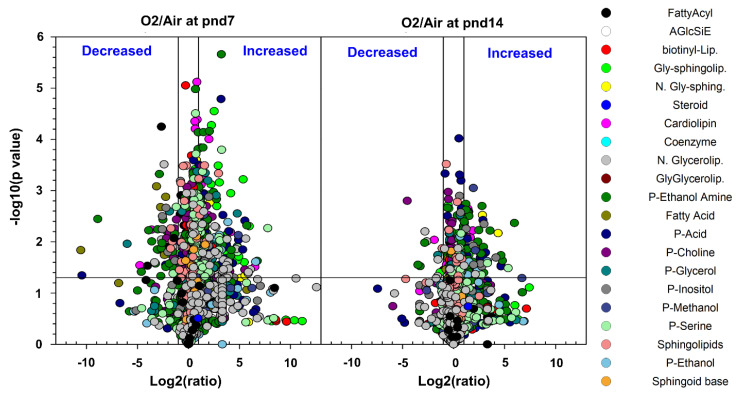
Volcano plots of differentially abundant lipids in mice exposed to hyperoxia as neonates. C57BL/6J mice (<12 h old) were exposed to hyperoxia (>95% O_2_) or room air (21% O_2_) for 3 days, and allowed to recover in room air until pnd7 or pnd14. Lung lipidome was measured using LC–MS. The -log10 *t*-test *p* values were plotted against the log2 ratio in mole fraction abundances of the phospholipid molecular species. Each plot has two areas, in the upper right and upper left, which represent 24-fold increase or decrease, respectively, in hyperoxia-exposed mice compared to the air group at both pnd7 and pnd14 (*p* < 0.05). Each lipid is represented as a circle, with color indicating the class. *n* = 6 mice per group. P-acid: phosphatidic acid; P-choline: phosphatidylcholine; P-glycerol: phosphatidylglycerol; P-inositol: phosphatidylinositol; P-methanol: phosphatidylmethanol; P-serine: phosphatidylserine; P-ethanol: phosphatidylethanol.

We next created Volcano plots using lipidX, which showed the changes in lung lipid species between hyperoxia- and air-exposed groups (Figure 1). Each plot had two areas, in the upper right and upper left, which represented a 24-fold increase or decrease, respectively, in hyperoxia-exposed compared to air-exposed mice at pnd7 or pnd14 (*p* < 0.05). In addition, there was a smaller number of lipid species that were decreased in hyperoxia-exposed mouse lungs at pnd14 compared to those at pnd7 (Figure 1). These results represent one of the largest lipidome datasets during alveolarization, and suggest that hyperoxic exposure alters lipid species in neonatal mouse lungs.

### 2.2. Neonatal Hyperoxia-Induced Alteration in Specific Lipid Categories

#### 2.2.1. Glycerophospholipids

**Figure 2:**
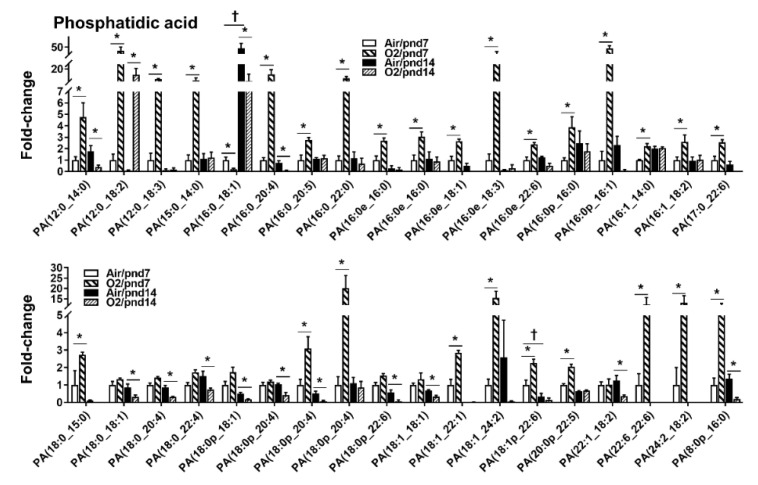
Compositional changes of lung PA species in mice exposed to hyperoxia as neonates. PA species in mouse lungs with 2-fold change and *p* < 0.05 between air and hyperoxia groups at pnd7 and pnd14 were listed. *n* = 6. * *p* < 0.05 vs. corresponding air; ^†^ *p* < 0.05 vs. air/pnd7.

Glycerophospholipids are fatty acid diglycerides with a phosphatidyl ester (e.g., choline, ethanolamine, serine, or inositol) attached to the terminal carbon, which are present in the highest amounts in the membranes of all cells. They are initially synthesized by the addition or transfer of the fatty acid chains to the glycerol backbone to form the first intermediate, lysophosphatidic acid (LPA) (Figure S1A). A total of 1561 glycerophospholipid species were identified in the air group at pnd7, spanning 10 subclasses (Table 1, Figure S2). Among 151 PAs, there were 37 PA species (24.5%) that were increased or decreased by hyperoxic exposure (Figure 2). At pnd7, there were 25 PA species showing a more than 2-fold increase by hyperoxic exposure. The top five increased PA species were PA(12:0_18:2), PA(16:0e_18:3), PA(16:0p_16:1), PA(18:0p_20:4), and PA(18:1_24:2). Only an identification of PA(16:0_18:1) showed more than a 2-fold decrease by hyperoxia at pnd7. At pnd14, only PA(12:0_18:2) was increased (≥5-fold) by hyperoxic exposure. In contrast, the levels of 14 PA species were significantly decreased by hyperoxic exposure at pnd14.

**Figure 3:**
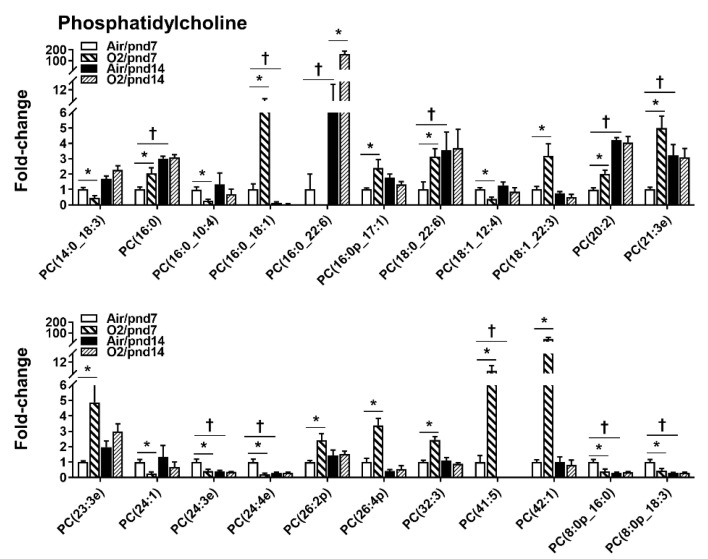
Compositional changes of lung PC species in mice exposed to hyperoxia as neonates. PC species in mouse lungs with 2-fold change and *p* < 0.05 between air and hyperoxia groups at pnd7 and pnd14 were listed. *n* = 6. * *p* < 0.05 vs. corresponding air; ^†^ *p* < 0.05 vs. air/pnd7.

A total of 22 PC species were altered by hyperoxic exposure among 346 species (Figure 3). Neonatal hyperoxia increased levels of 13 PC species; the top 5 were PC(16:0_18:1), PC(16:0_18:1), PC(21:3e), PC(41:5), and PC(42:1) at pnd7. Levels of PC(14:0_18:3), PC(16:0_10:0), PC(18:1_12:4), PC(24:1), PC(24:3e), PC(24:4e), PC(8:0p_16:0), and PC(8:0p_18:3) were reduced by hyperoxic exposure at pnd7. At pnd14, we only found one PC species, PC(16:0_22:6), that showed a 23-fold increase in the hyperoxia-exposed mouse lungs.

**Figure 4:**
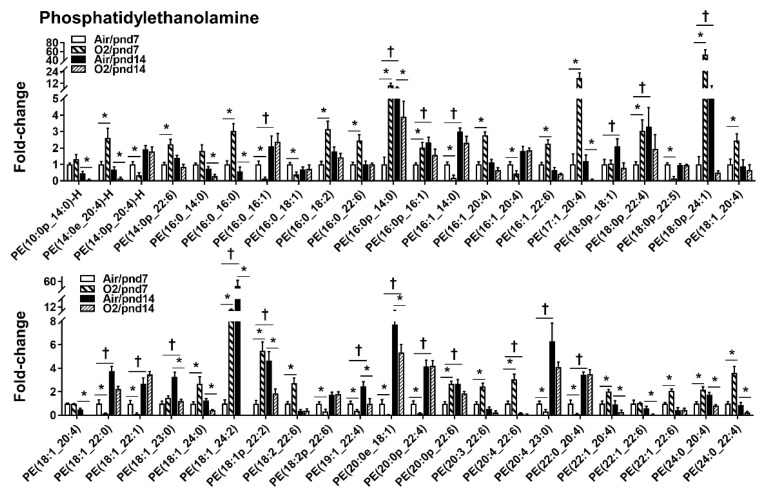

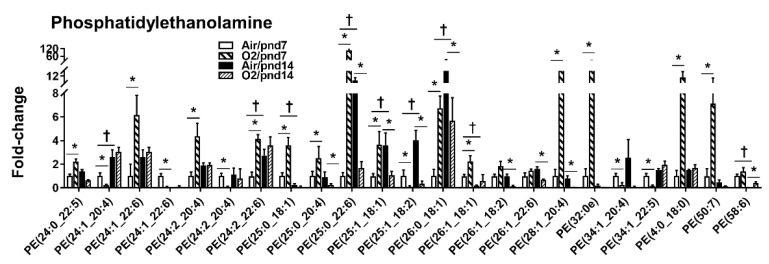
Compositional changes of lung PE species in mice exposed to hyperoxia as neonates. PE species in mouse lungs with 2-fold change and *p* < 0.05 between air and hyperoxia groups at pnd7 and pnd14 were listed. *n* = 6. * *p* < 0.05 vs. corresponding air; ^†^ *p* < 0.05 vs. air/pnd7.

Among 413 PEs, neonatal hyperoxia caused a 2-fold increase in 39 PE species, and a 5-fold increase in 13 PEs in mice at pnd7 (Figure 4). The levels of 20 PEs were significantly reduced by hyperoxia at pnd7. At pnd14, we found that there were no PE species that were increased by hyperoxic exposure. Levels of PE species (i.e., PE(18:0p_24:1), PE(18:1_24:2), PE(25:0_22:6), PE(25:1_18:1), and PE(25:1_18:2)) were significantly reduced by hyperoxia at pnd14.

**Figure 5:**
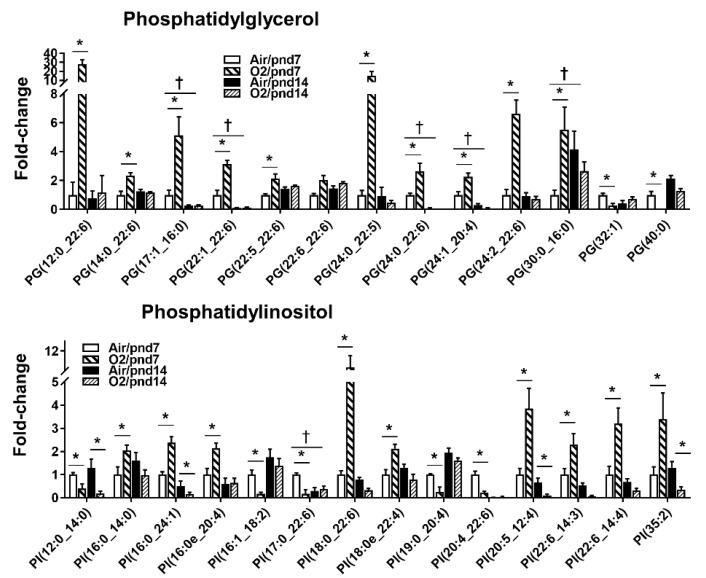
Compositional changes of lung PG and PI species in mice exposed to hyperoxia as neonates. PG and PI species in mouse lungs with 2-fold change and *p* < 0.05 between air and hyperoxia groups at pnd7 and pnd14 were listed. *n* = 6. * *p* < 0.05 vs. corresponding air; ^†^ *p* < 0.05 vs. air/pnd7.

At pnd7, we found that hyperoxic exposure increased levels of 11 PG species among 147 PG species (Figure 5). The levels of PG(32:1) and PG(40:0) were reduced by hyperoxia at pnd7. Neonatal hyperoxic exposure did not alter the levels of PE species in mouse lungs at pnd14.

Among 120 PI species, 8 of them were increased by hyperoxic exposure, and the top 5 were PI(18:0_22:6), PI(25:0_12:4), PI(22:6_14:3), PI(22:6_14:4), and PI(35:2) at pnd7 (Figure 5). The levels of PI(12:0_14:0), PI(16:1_18:2), PI(17:0_22:6), PI(19:0_20:4), and PI(20:4_22:6) were significantly reduced by hyperoxic exposure at pnd7. No PI species were significantly increased by neonatal hyperoxia at pnd14. The levels of eight PI species were significantly reduced by hyperoxia at pnd14.

**Figure 6:**
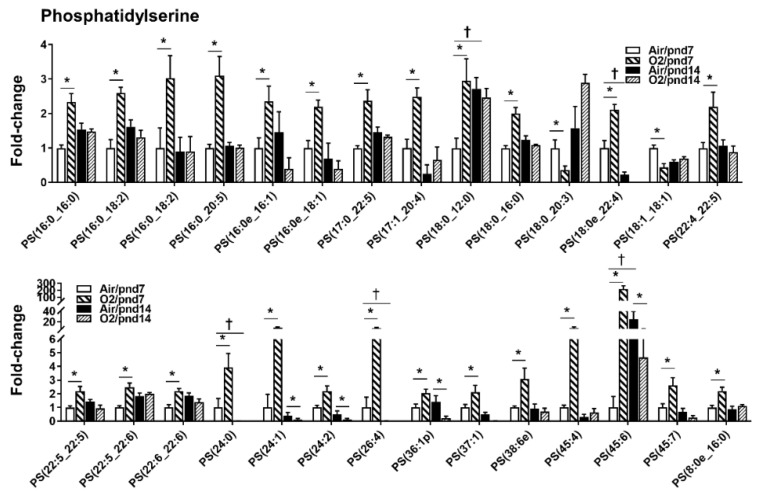
Compositional changes of lung PS species in mice exposed to hyperoxia as neonates. PS species in mouse lungs with 2-fold change and *p* < 0.05 between air and hyperoxia groups at pnd7 and pnd14 were listed. *n* = 6. * *p* < 0.05 vs. corresponding air; ^†^ *p* < 0.05 vs. air/pnd7.

Among 170 PS species, 25 of them (14.7%) were increased by hyperoxic exposure at pnd7 (Figure 6). The top five of them were PS(24:0), PS(24:1), PS(26:4), PS(45:4), and PS(45:6). There was one PS species (i.e., PS(18:0_20:3)) that was reduced by hyperoxia at pnd7. At pnd14, the level of PS(36:1p) was reduced, whereas there were no increases in any PS species by hyperoxic exposure.

**Figure 7:**
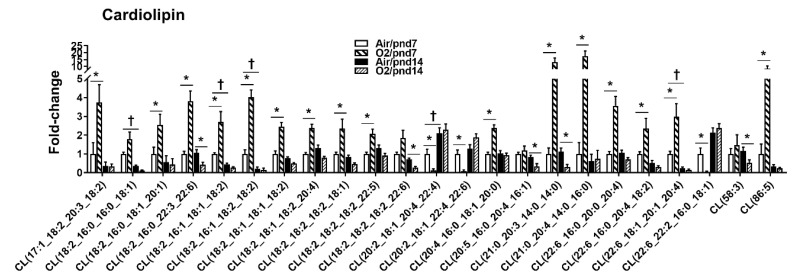
Compositional changes of lung CL species in mice exposed to hyperoxia as neonates. CL species in mouse lungs with 2-fold change and *p* < 0.05 between air and hyperoxia groups at pnd7 and pnd14 were listed. *n* = 6. * *p* < 0.05 vs. corresponding air; ^†^ *p* < 0.05 vs. air/pnd7.

At pnd7, 17 CL species (22.9%) were increased in mouse lungs among 74 CL species by hyperoxic exposure (Figure 7). The top five increased CL species were CL(18:2_16:0_22:3_22:6), CL(18:2_18:1_18:1_18:2), CL(21:0_20:3_14:0_14:0), CL(21:0_20:4_14:0_16:0), and CL(86:5). Levels of CL(20:2_18:1_20:4_22:4), CL(20:2_18:1_22:4_22:6), and CL(22:6_22:2_16:0_18:1) were reduced by hyperoxic exposure at pnd7. There were no CLs that were augmented by hyperoxia at pnd14. A few CLs were reduced by hyperoxic exposure at pnd14, which included CL(18:2_16:0_16:0_18:1), CL(18:2_16:0_22:3_22:6), CL(18:2_18:2_18:2_18:1), CL(18:2_18:2_18:2_22:6), CL(21:0_20:3_14:0_14:0), and CL(58:3).

#### 2.2.2. Lysophospholipids

Lysophospholipids can be generated from phospholipase-mediated hydrolyzation of phospholipids and sphingolipids (Figure S1B). Among 14 LPA species, levels of LPA(20:4), and LPA(24:0) were increased, while LPA (18:3) was reduced by hyperoxic exposure at pnd7 (Figure S3). Hyperoxic exposure did not alter any LPA species in mouse lungs at pn14.

Among 92 lysophosphatidylcholine (LPC) species, levels of LPC(16:0), LPC(18:1), and LPC(22:6) were increased, whereas levels of LPC(20:0) and LPC(22:3) were reduced by hyperoxic exposure at pnd7 (Figure S3). The levels of LPC(18:1) and LPC(20:5) were significantly reduced by hyperoxia at pnd14.

A total of 51 lysophosphatidylethanolamines (LPE) were identified in mouse lungs. Levels of LPE(16:0), LPE(18:0e), LPE(18:1), LPE(18:2), and LPE(20:0p) were increased, while LPE(16:0p) was reduced by hyperoxic exposure at pnd7 (Figure S3). At pnd14, hyperoxic exposure reduced levels of LPE(16:0p) and LPE(18:0e) in mouse lungs.

Among 16 lysophosphatidylglycerol (LPG) identified, 7 of them (LPG(16:0), LPG(18:3), LPG(20:3), LPG(22:0), LPG(22:4), LPG(24:0), and LPG(24:2)) were significantly increased by hyperoxia at pnd7 (Figure S3). Hyperoxic exposure did not alter levels of LPG in mouse lungs at pnd14.

Among eight lysophosphatidylserine (LPS) species, levels of LPS(22:0), LPS(24:0), and LPS(24:1) were increased by hyperoxic exposure at pnd7. There were no changes in LPS species in hyperoxia-exposed mice at pnd14 (Figure S3).

#### 2.2.3. Sphingolipids

**Figure 8:**
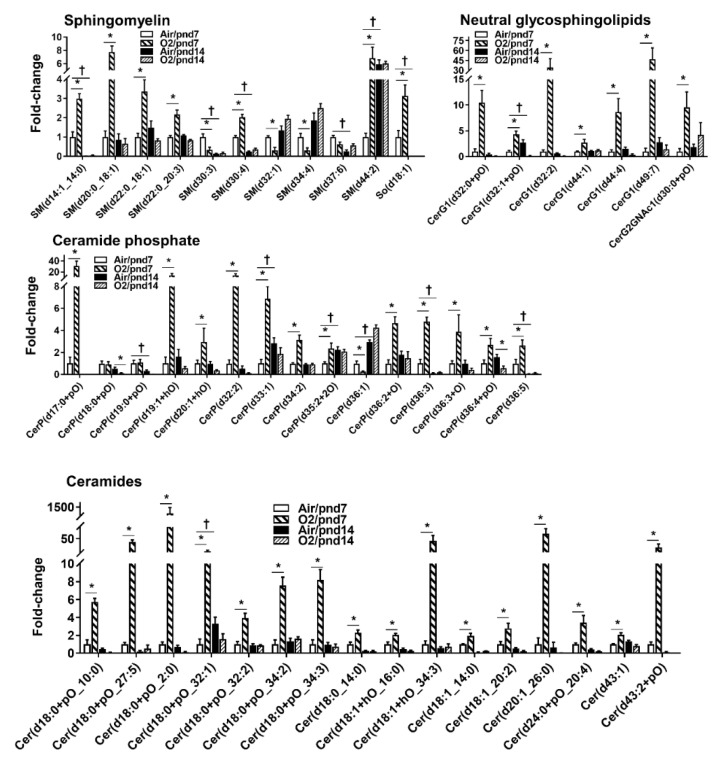
Compositional changes of lung sphingolipid species in mice exposed to hyperoxia as neonates. SM, CerP, neutral glycosphingolipid, and Cer species in mouse lungs with 2-fold change and *p* < 0.05 between air and hyperoxia groups at pnd7 and pnd14 were listed. *n* = 6. * *p* < 0.05 vs. corresponding air; ^†^ *p* < 0.05 vs. air/pnd7.

Bioactive sphingolipids include sphingomyelin, ceramide, sphingosine, sphingosine-1-phosphate (S1P), and ceramide-1-phosphate (Figure S4). Within the sphingolipid category, 322 lipid species were identified, which covered four sphingolipid subclasses (Table 1, Figure S5). Among 88 sphingomyelin (SM) species, 6 were significantly increased, while 3 were reduced in hyperoxia-exposed mice at pnd7 compared to the air group (Figure 8). There was one SM species, SM(d37:6), that was increased, whereas no decrease in SM species was found in hyperoxia-exposed mice at pnd14.

A total of 110 ceramide species were identified in mouse lungs at pnd7. Hyperoxic exposure increased levels of 16 ceramide species (14.5%), while no ceramides were reduced by hyperoxic exposure at pnd7 (Figure 8). Interestingly, hyperoxic exposure did not alter any ceramide species in mouse lungs at pnd14. We noticed that levels of ceramides were low in normoxia-exposed mice at pnd14 compared to pnd7.

Among 57 ceramide phosphate (CerP) species, 12 CerP were increased by hyperoxic exposure at pnd7. One CerP species (i.e., CerP(d36:1)) was reduced by hyperoxia at pnd7 (Figure 8). Hyperoxic exposure reduced levels of CerP(d18:0+pO), CerP(d19:0+pO), CerP(d19:1+hO), CerP(d20:1+hO), CerP(d32:2), CerP(d36:3+O), and CerP(d36:4+pO) in mouse lungs at pnd14.

There were 59 neutral glycosphingolipids that were identified in normoxia-exposed mice at pnd7 (Figure 8). Seven of them were significantly increased, while no decrease in neutral glycosphingolipids were found in hyperoxia-exposed mice at pnd7. At pnd14, no increases in neutral glycosphingolipids were found, while levels of CerG1(d32:1+pO), CerG1(d32:2), and CerG1(d44:4) were significantly reduced by hyperoxic exposure.

#### 2.2.4. Glycerolipids

A total of 150 neutral glycerolipids were identified, and this included triacylglycerol (TG), diacylglycerol (DG), and monoacylglycerol (MG) subclasses. There were 25 TG species (23.8%) that were altered by hyperoxia either at pnd7 or pnd14. Among them, we noticed 17 TG species that were increased at pnd14 compared to pnd7 in normoxic conditions (Figure S6). Levels of 21 TG species were significantly increased by hyperoxia at pnd7. The top five increased TG species were TG(10:0_18:2_18:3), TG(14:0_20:5_22:6), TG(16:0_20:5_22:6), TG(20:5_22:6_22:6), and TG(60:7p). Hyperoxic exposure did not reduce any TG species in mouse lungs at pnd7. At pnd14, hyperoxic exposure did not increase any TG species, but reduced levels of TG(20:5_22:6_22:6) in mouse lungs.

A total of 31 diglyceride (DG) species were identified in mouse lungs at pnd7. Eight of them were increased by hyperoxic exposure at pnd7, which were DG(10:0_18:2), DG(16:0_22:6), DG(18:0_20:3), DG(18:1_22:1), DG(35:1_16:0), DG(51:4), DG(55:6), and DG(56:5) (Figure S6). Levels of DG(16:0_16:0), DG(16:0_16:1), and DG(16:0_18:2) were reduced in hyperoxia-exposed mice at pnd7. At pnd14, there was only one DG(10:0_18:2) that was increased by hyperoxic exposure.

#### 2.2.5. Fatty Acids

**Figure 9:**
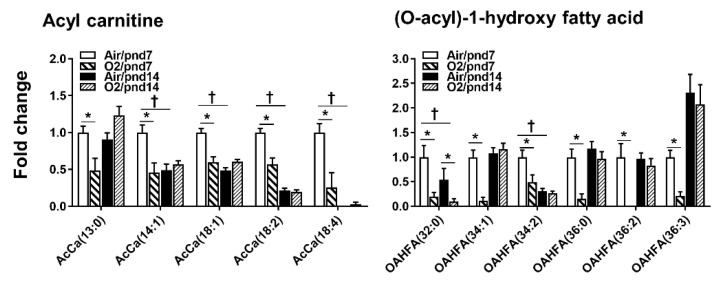
Compositional changes of lung AcCa and OAHFA species in mice exposed to hyperoxia as neonates. AcCa and OAHFA species in mouse lungs with 2-fold change and *p* < 0.05 between air and hyperoxia groups at pnd7 and pnd14 were listed. *n* = 6. * *p* < 0.05 vs. corresponding air; ^†^ *p* < 0.05 vs. air/pnd7.

Among 36 acyl carnitine (AcCa) species, hyperoxic exposure reduced levels of acyl carnitine, including AcCa(13:0), AcCa(14:1), and AcCa(18:4), whereas no increase was observed in acyl carnitine in mouse lungs at pnd7 (Figure 9). At pnd14, hyperoxic exposure did not alter levels of acyl carnitine in mouse lungs. Levels of AcCa(14:1), AcCa(18:1), AcCa(18:2), and AcCa(18:4) were reduced in normoxia-exposed mice at pnd14 compared to those at pnd7 (Figure 9).

There were 14 (O-acyl)-1-hydroxy fatty acid (OAHFA) species that were detected in mouse lungs. Hyperoxic exposure reduced levels of 6 OAHFA species in mouse lungs at pnd7 (Figure 9). At pnd14, we did not observe any changes in OAHFA species except that levels of OAHFA(32:0) were reduced by hyperoxic exposure, suggesting that the OAHFA may participate in hyperoxia-induced lung injury.

### 2.3. Neonatal Hyperoxia Dynamically Altered Metabolites for Fatty Acid Synthesis and Oxidation

**Figure 10:**
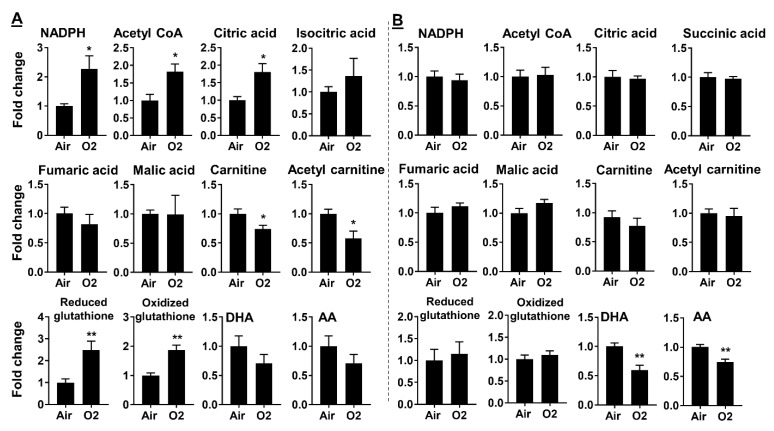
Neonatal hyperoxia dynamically altered metabolites for fatty acid synthesis and oxidation in mouse lungs. C57BL/6J mice (<12 h old) were exposed to hyperoxia (>95% O_2_) or room air (21% O_2_) for 3 days, and were allowed to recover in room air until pnd7 (**A**) or pnd14 (**B**). Lung metabolomics were measured using LC–MS. Data are expressed as mean ± SEM. *n* = 6. * *p* < 0.05, ** *p* < 0.01 vs. air.

Neonatal hyperoxia increased levels of NADPH, acetyl CoA, and citric acid in mouse lungs at pnd7 (Figure 10A). This response was not observed in mouse lungs at pnd14 (Figure 10B). There were no changes in isocitric acid, succinic acid, fumaric acid, or malic acid in mice exposed to hyperoxia as neonates. Neonatal hyperoxia reduced levels of carnitine and acetyl carnitine in mouse lungs at pnd7 but not at pnd14. At pnd7, neonatal hyperoxia did not alter levels of docosahexaenoic acid (DHA) and arachidonic acid (AA) in mouse lungs (Figure 10A). Their levels were significantly reduced at pnd14 in mice exposed to hyperoxia as neonates (Figure 10B). These results suggest that neonatal hyperoxia increases fatty acid synthesis but reduces fatty acid oxidation at pnd7.

### 2.4. Neonatal Hyperoxia Increased Oxidative Stress in Mouse Lungs at pnd7 but Not pnd14

**Figure 11:**
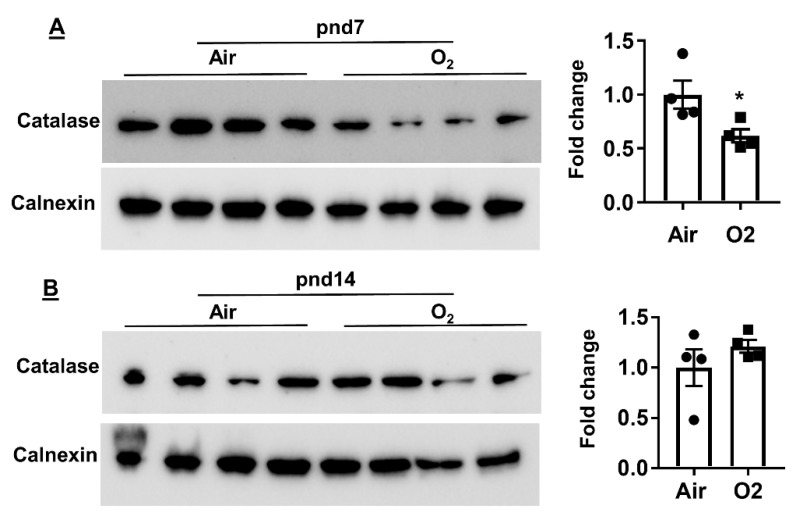
Protein levels of lung catalase in mice exposed to hyperoxia as neonates. C57BL/6J mice (<12 h old) were exposed to hyperoxia (>95% O_2_) or room air (21% O_2_) for 3 days, and were allowed to recover in room air until pnd7 or pnd14. Western blot was performed to determine the levels of catalase in lungs at both pnd7 (**A**) and pnd14 (**B**). Data are expressed as mean ± SEM. *n* = 4. * *p* < 0.05 vs. air.

Hyperoxia-mediated active oxygen species are chemically very aggressive, which can cause lipid peroxidation, leading to severe damage to the membranes [15]. Therefore, we determined levels of lung antioxidants, including glutathione and catalase, in mice exposed to hyperoxia as neonates. As shown in Figure 10, neonatal hyperoxia increased oxidized glutathione in mouse lungs at pnd7 but not pnd14. Furthermore, protein levels of lung catalase were reduced at pnd7 but not at pnd14 in mice exposed to hyperoxia as neonates (Figure 11). These results suggest that neonatal hyperoxia causes oxidative stress in mouse lungs at pnd7.

## 3. Discussion

There are a few studies showing compositional changes in the lipidome during postnatal lung development in mice and humans [11,12,16,17,18]. Whether neonatal hyperoxia alters this lung lipid profile in mice remains unclear. Here, we utilized LC–MS to measure stage-specific alterations of individual lung lipid species during the peak stage of alveolarization in response to exposure to hyperoxia in the neonatal period. We found that certain species of glycerophospholipid and TG were increased at pnd14 compared to those at pnd7 in normoxic conditions. Three-day hyperoxic exposure in newborn mice significantly altered the lipidome in lungs at pnd7, but this returned to normal at pnd14. These results suggest that lipids play important roles in lung alveolarization, and this was altered by hyperoxic exposure

The glycerophospholipids account for about 80% of pulmonary surfactant. In general, the amounts of lung glycerophospholipids (PC, PE, PG, PI, and PS) are significantly reduced in adult mice compared to those in neonatal mice (pnd7 and pnd14) [11]. Levels of certain glycerophospholipid species are also altered from the saccular phase to the alveolar stages [11,12]. This is corroborated by increased glycerophospholipids of pulmonary tissue at pnd14 compared to those at pnd7. These glycerophospholipids may be utilized for pulmonary surfactant synthesis by proliferating type II cells during the expansion of the lung interstitium [19]. It is not clear whether neonatal hyperoxia causes an early and compensatory increase in glycerophospholipid in mouse lungs.

LPC (16:0) and LPC (22:6) were increased at pnd14 compared to those at pnd7 in normoxic exposure, which agrees with their continuous increase during lung development [12]. Neonatal hyperoxia has been shown to increase the levels of phospholipases A2 in mouse lungs [20]. This may be one of the reasons for increased glycerophospholipid hydrolysis to lysophospholipids by phospholipases A2 in hyperoxia-exposed mice at pnd7.

CL localizes in the inner mitochondrial membrane. It is required for mitochondrial metabolism by interacting mitochondrial proteins/enzymes and maintaining inner membrane fluidity and osmotic stability [21]. Abnormally increased CL is able to cause a release of cytochrome C from mitochondria into the cytosol, leading to apoptosis [22]. In the lungs, high levels of CL could disrupt the surface tension by reducing the capability of pulmonary surfactant, resulting in reduced lung compliance [23]. In adult mice, exposure to hyperoxia does not alter amount of CL or PS, but causes their oxidation in the lungs [24]. Although neonatal hyperoxia increased oxidative stress in lungs at pnd7, it remains unclear whether oxidation/peroxidation of CL or PS occurs in neonatal hyperoxia-exposed lungs.

Sphingolipids modulate cell fates during lung development and the development of lung diseases [25,26]. Ceramides and sphingosine cause apoptosis and inflammatory response, while S1P facilitates proliferation and differentiation as well as protects against apoptosis and ventilation-induced lung injury [27]. In agreement with our findings, neonatal hyperoxia for four weeks augments sphingomyelin species (SM16:0, SM18:0, SM24:0, and SM24:1), long chain ceramides (Cer16:0 and Cer18:0), and very long chain ceramides (Cer24:0 and Cer24:1) in bronchoalveolar lavage fluid from mice [28], and ceramide is increased in tracheal aspirates of preterm infants [9]. This may result in increased apoptosis with neonatal exposure to hyperoxia [2,29], despite there being no lung inflammatory responses observed in our 3-day hyperoxia-exposed mice (data not shown). Increased ceramide may be due to either increased hydrolysis of sphingomyelin or de novo synthesis in response to hyperoxic exposure [2].

Glycosphingolipids (GSL) consist of a ceramide backbone with a glycan moiety, which can be grouped into a galactosylated (LacCer) or glucosylated ceramide (GlcCer, CerG). This diversity of GSLs is well studied during embryogenesis and at specific developmental stages [30]. A dramatic change in the GSL composition in mouse lungs is observed between pnd1 and pnd7 [16]. GlcCer and LacCer, as receptors for pulmonary surfactant protein SP-A, are the predominant neutral glycosphingolipids prenatally and at pnd1, while levels of globo- and ganglio-series GSL dramatically increased after pnd7, reaching maxima at pnd14 and pnd21 [16]. This is corroborated by our findings that CerG1(d32:1+pO) and CerG1(d49:7) were increased in mouse lungs at pnd14 compared to pnd7. Further study is required to determine whether hyperoxia-induced increase in neutral glycosphingolipids is compensatory for binding to SP-A in lungs of rodents exposed to hyperoxia as neonates [31].

Consistent with our findings, lung TG content gradually increases after birth, peaking at pnd14 [11,17]. Hyperoxic exposure increased the levels of TG and DG in mouse lungs at pnd7, which may be one of the mechanisms for hypertriglyceridemia in infants with BPD [32].

Cytosol citrate can be catalyzed by acetyl-CoA ligase to generate acetyl CoA, which is utilized for the synthesis of fatty acids along with NADPH [33]. Carnitine palmitoyltransferase 1 catalyzes the transfer of the acyl group of long-chain fatty acids from coenzyme A to carnitine to form acyl carnitine for mitochondrial transport and subsequent fatty acid oxidation. Neonatal hyperoxia increased levels of NADPH, acetyl CoA and citrate acid, but reduced carnitine, acyl carnitine, and carnitine palmitoyltransferase 1 in mouse lung at pnd7 but not at pnd14 [2], suggesting increased fatty acid synthesis and reduced fatty acid oxidation in hyperoxia-exposed mouse lungs at the earlier time-point. This may contribute to the augmented synthesis of the lung lipidome observed in mice exposed to hyperoxia as neonates at pnd7. CL is required for carnitine/acyl carnitine translocase activity that is responsible for transport of acyl carnitine into the mitochondrial matrix for β-oxidation [21,34]. Thus, at pnd7, hyperoxia-induced reduction in acyl carnitine may be due to its increased mitochondrial transport. DHA is an omega-3 fatty acid, which suppresses apoptosis in the lungs of mice perinatally exposed to lipopolysaccharide/hyperoxia [35]. Furthermore, decreased postnatal DHA and AA levels are associated with morbidities in premature infants [36]. Neonatal hyperoxia decreased both DHA and AA levels in mouse lungs at pnd14 but not at pnd7. Further study is required to determine whether neonatal hyperoxia causes persistent changes in DHA and AA in adult mice and their contribution to alveolar simplification and dysregulated vascular development.

Although neonatal hyperoxia induced transient changes in lipidome, increased oxidative stress-mediated lipid peroxidation may cause sustained and persistent injurious effects on lung alveolarization and vascularization in neonates. We did not detect lung lipidome at another time-point (e.g., pnd30), which would more clearly trace the trend of specific lipid species from lung alveolarization to maturation after neonatal hyperoxia. Further study is required to differentiate these lung lipidomic changes intracellularly and extracellularly as well as cell-specific localization in mice exposed to hyperoxia as neonates.

In conclusion, our study provides an extensive lipid profile of whole mouse lung tissue, including less abundant lipid species and neutral lipid components, across alveolarization under normoxic conditions. This work also demonstrates that neonatal hyperoxic exposure caused a transient alteration of this lipid profile. These findings may enhance the understanding of lipid alterations during lung development as well as how dysregulated lipid metabolism plays a role in the pathogenesis of hyperoxic lung injury. The new lipidomic signature observed in this study could also provide potential therapeutic targets for hyperoxic lung injury.

## 4. Methods

### 4.1. Hyperoxic Exposure

Newborn C57BL/6J mice (<12 h old) along with their mothers were exposed to room air or hyperoxia (>95% O_2_) for 72 h in an A-chamber (BioSpherix, Parish, NY, USA) [2]. The dams were switched every 24 h between room air and hyperoxia to avoid injury. The pups were allowed to recover in room air until pnd7 or pnd14. There were 6 mice per group. All animal experiments were reviewed and approved by the Institutional Animal Care and Use Committee of Brown University.

### 4.2. Biphasic Extraction of Lung Tissues for Lipidomics and Metabolomics

Approximately 50 mg of lung tissue was homogenized with 2 mL of chilled methanol (MX0486-1, Sigma, St. Louis, MO, USA). These mixtures were combined with 4 mL of HPLC grade chloroform (A452-1, Sigma, St. Louis, MO, USA) on ice. The mixtures were vortexed for 3 min, and then, combined with 2 mL of HPLC grade water (WX0001-1, Sigma, St. Louis, MO, USA). The samples were vortexed and centrifuged at 3000 rpm for 10 min at 4 °C. The bottom layer (chloroform phase) was collected for lipidomics analysis, while the top layer (aqueous phase) was collected for untargeted metabolomics analysis.

### 4.3. Lipidomics Analysis

The chloroform phase was used to detect the lipidome using LC–MS on an Orbitrap Exactive (Thermo Scientific, Waltham, MA, USA) in line with an Ultimate 3000 LC (Thermo Scientific, Waltham, MA, USA) [37]. Each sample was analyzed in positive and negative modes, in top 5 automatic data-dependent MS/MS mode. Column hardware consisted of a Biobond C4 column (4.6 × 50 mm, 5 μm, Dikma Technologies, Foothill Ranch, CA, USA). Flow rate was set to 100 μL/min for 5 min with 0% mobile phase B, then, switched to 400 μL/min for 50 min, with a linear gradient of mobile phase B from 20% to 100%. The column was then washed at 500 μL/min for 8 min at 100% mobile phase B before being re-equilibrated for 7 min at 0% mobile phase B and 500 μL/min. For positive mode runs, buffers consisted, for mobile phase A, of 5 mM ammonium formate, 0.1% formic acid, and 5% methanol in water, and, for mobile phase B, of 5 mM ammonium formate, 0.1% formic acid, 5% water, and 35% methanol in Isopropanol. For negative runs, buffers consisted, for mobile phase A, of 0.03% ammonium hydroxide and 5% methanol in water, and, for mobile phase B, of 0.03% ammonium hydroxide, 5% water, and 35% methanol in isopropanol. Each of the spectra for each lipid identified with Lipidsearch^©^ software (version 4.1.16, Mitsui Knowledge Industry, University of Tokyo) was manually examined for the presence of the head group characteristic fragment, and, if present, for the side chain’s fragment. Each ID was based on the fragments founds in the literature and compiled in the Lipidsearch database. Intensity signals are reported as area of the parent ion. Integrations and peak quality were curated manually before exporting and analyzing the data in Microsoft Excel.

Since the number of samples in the runs were low, the instrument sensitivity did not change significantly, and no repeated quality control sample injections were needed. The instrument was running a constant internal calibration with lock masses for mass accuracy, insuring no drift in the mass accuracy over the span of the sample runs.

### 4.4. Metabolomics Assay

The top layer (aqueous phase) was collected for untargeted metabolomics analysis using a Thermo Fisher Ultimate 3000 LC coupled with a Q-Exactive Plus mass spectrometer. Five microliters of each sample were injected on a Zic-pHILIC Column (150 × 2.1 mm, 5 µm particles, EMD Millipore, Burlington, MA, USA). The mobile phases were (A) 20 mM ammonium carbonate in 0.1% ammonium hydroxide and (B) acetonitrile 97% in water. The gradient conditions were as follows: 100% B at 0 min, 40% B at 20 min, 0% B at 30 min for 5 min, then back to 100% B in 5 min, followed by 10 min of re-equilibration. Full ms spectra were acquired in switching polarity at 70,000 resolution, covering a range of mz 66 to 1000. Compound discoverer 3.0 (CD, Thermo Fisher, Waltham, MA, USA) was used to generate a list of features (mz and retention time) found in a pool sample (pool of all samples). This list was used as an inclusion list for MS/MS runs in positive and negative ion modes separately. Using CD, the features which were selected for MS/MS in the first MS/MS run were then removed from the inclusion list and the MS/MS experiments were repeated with the new list. By repeating this process four times, we were able to obtain MS2 data for most of the features detected by CD. All the data were then combined and analyzed in CD. Likely elemental compositions were computed based on the accurate mass and isotope pattern, and mzCloud msms spectra database, a local MzVault database, and chemspider libraries were searched to identify possible candidates. Each compound was then manually curated to ensure proper integration and the accuracy of the mz cloud/mz vault hits was also checked. Compounds with high quality MS/MS library match were assigned the name of their match. Intensity signals were reported as normalized area of the parent ion.

### 4.5. Measurement of Protein by Western Blot

Lung tissues were homogenized using RIPA buffer, and 10 μg proteins were separated on a NuPAGE^TM^ 4–12% Bis-Tris protein gel (Invitrogen, Carlsbad, CA, USA) followed by protein transferring onto nitrocellulose membranes. The blots were blocked for 1 h at room temperature with 5% BSA, and then, probed with primary antibodies against catalase (Cat#: ab16731, Abcam, Cambridge, UK) and calnexin (ADI-SPA-860-F, Enzo Life Science, Nassau County, NY, USA) at 4 °C overnight. Protein levels were detected using secondary antibodies in 5% BSA in PBS containing 0.1% Tween (*v*/*v*) 20 for 1 h) linked to horseradish peroxidase (Vector Laboratories, Burlingame, CA, USA), and bound complexes were detected by the ChemiDoc^TM^ Touch Imaging System (BIO-RAD, Hercules, CA, USA) using the enhanced chemiluminescence method (Millipore, Burlington, MA, USA).

### 4.6. Statistical Analysis

The results were expressed as mean ± SEM. Statistical analyses were performed using GraphPad Prism 7. The unpaired two sample t-test was used for detecting statistical significance of the differences between means of two groups after checking the normality of data. The statistical significance of the differences among groups was evaluated by using two-way ANOVA for overall significance, followed by the Tukey–Kramer test. Statistical significance was considered existing when *p* < 0.05. The lipid species from the lipidomics analysis were present in histograms if (1) mean values of the hyperoxia group had 2-fold changes compared to air group; and (2) *p* values were less than 0.05.

## 5. Conclusions

The lung lipidome changes throughout alveolarization. Neonatal hyperoxia alters the lung lipidome, which may contribute to alveolar simplification and dysregulated vascular development.

## Figures and Tables

**Table 1:** Numbers of lipids detected in lung homogenates.

Class of Lipids	Subclass of Lipids	pnd7		pnd14	
		Air	O2	Air	O2
Glycerophospholipids	Phosphatidic acid (PA)	151	154	154	152
	Phosphatidylcholine (PC)	347	346	345	343
	Phosphatidylethanolamine (PE)	413	413	412	409
	Phosphatidylethanol	52	57	57	58
	Phosphatidylglycerol (PG)	147	146	149	149
	Phosphatidylinositol (PI)	120	120	113	115
	Phosphatidylmethanol (PMe)	35	35	35	34
	Phosphatidylserine (PS)	170	168	169	164
	Phosphatidylethanol (PEth)	52	57	57	58
	Cardiolipin (CL)	74	74	75	75
Sphingolipids	Sphingolipids	88	88	88	88
	Sphingoid base	12	12	11	11
	Glycosphingolipids	163	167	163	163
	Neutral glycosphingolipids	59	60	57	57
Glycerolipids	Neutral glycerolipid (e.g., TG, DG, and MG)	150	163	156	155
	Glycoglycerolipid	4	5	5	4
Fatty acyls	Fatty acid	19	20	19	19
	Fatty acyl and other lipids	57	58	49	49
Sterol lipids	Steroid	4	6	4	4
Derivatized lipids		141	145	144	143

## Supplementary Materials

The following are available online at www.mdpi.com/xxx/s1-S6, Figure S1: Simplified schematic of synthesis and hydrolysis of glycerophospholipid, Figure S2: Lung glycerophospholipid species in mice exposed to hyperoxia as neonates, Figure S3: Compositional changes of lung glycerophospholipid species in mice exposed to hyperoxia as neonates, Figure S4: Sphingolipid metabolic pathway, Figure S5: Lung sphingolipid species in mice exposed to hyperoxia as neonates, Figure S6: Compositional changes of lung TG and DG species in mice exposed to hyperoxia as neonates, Table S1: All lung lipid species in mice exposed to hyperoxia as neonates.

## Abbreviations

AA	Arachidonic acid
AcCa	Acyl carnitine
BPD	Bronchopulmonary dysplasia
Cer	Ceramide
CerP	Ceramide phosphate
CL	Cardiolipin
DG	Diglyceride
DHA	Docosahexaenoic acid
LC–MS	Liquid chromatography–mass spectrometry
LPA	Lysophosphatidic acid
LPC	Lysophosphatidylcholine
LPE	Lysophosphatidylethanolamine
LPG	Lysophosphatidylglycerol
LPS	Lysophosphatidylserine
OAHFA	O-acyl-1-hydroxy fatty acid
Pnd	Postnatal day
PA	Phosphatidic acid
PC	Phosphatidylcholine
PE	Phosphatidylethanolamine
PG	Phosphatidylglycerol
PI	Phosphatidylinositol
PS	Phosphatidylserine
SM	Sphingomyelin
TG	Triglyceride
