# Supplementary material for: Hyperoxic Exposure Caused Lung Lipid Compositional Changes in Neonatal Mice
Source: Metabolites. 2020 Aug 21;10(9):340. doi: 10.3390/metabo10090340 (PMC7569933; doi:10.3390/metabo10090340)
Supplement: Supplementary file 1 [file metabolites-10-00340-s001.zip › metabolites-877697-supplementary-/metabolites-877697-Figure S.docx]

Hyperoxic Exposure Caused Lung Lipid Compositional Changes in Neonatal Mice

**Supplementary Figure Legends**

**
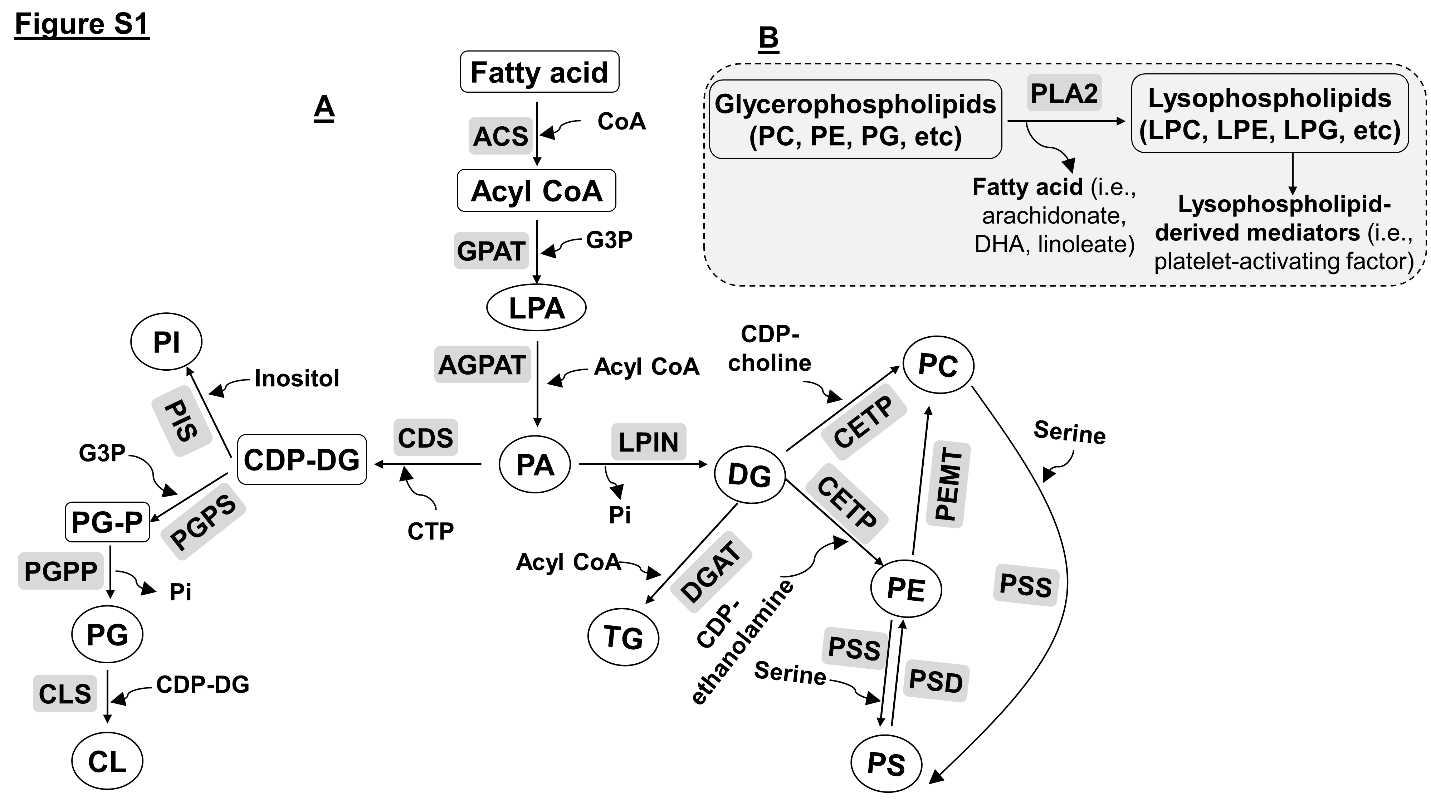
Figure 1. Simplified schematic of synthesis and hydrolysis of glycerophospholipid**

(A) Glycerophospholipids are synthesized from glycerol-3-phosphate (G3P) in a de novo pathway that initially produces phosphatidic acid (PA) and diacylglycerol (DG) or cytidine diphosphate-DAG (CDP-DG). (B) Generation of lysophospholipids from glycerophospholipids by phospholipases A2 (PLA2). ACS: acyl coa synthetase; AGPAT: acyl-CoA:1-acylglycerol-3-phosphate acyltransferase; CDS: CDP-DG synthase; CEPT: diacylglycerol choline/ethanolamine phosphotransferase; CL: cardiolipin; CLS: cardiolipin synthase; DGAT: diacylglycerol acyltransferase; LPA: lysophosphatidic acid; G3P: glycerol-3-phosphate; GPAT: Glycerol-3-phosphate acyltransferase; PC: phosphatidylcholine; PE: phosphatidylethanolamine; PEMT: phosphatidylethanolamine methyltransferase; PG: phosphatidylglycerol; PG-P: phosphatidylglycerophosphate; PGPP: phosphatidylglycerophosphate phosphatase; PGPS: phosphatidylglycerophosphate synthase; PI: phosphatidylinositol; PIS: phosphatidylinositol synthase; PS: phosphatidylserine; PSD: phosphatidylserine decarboxylase; PSS: phosphatidylserine synthase; TG: triacylglycerol.


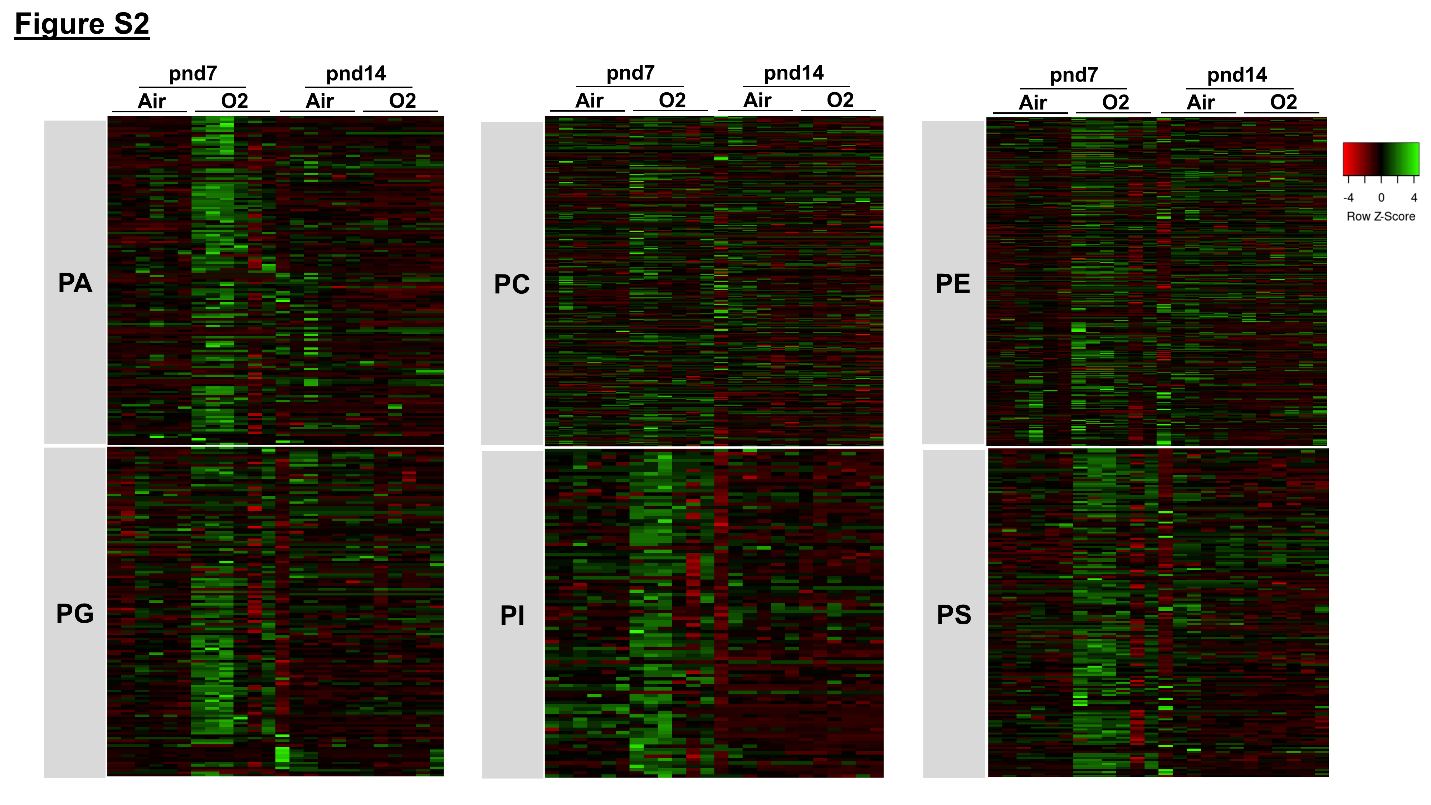
**Figure S2. Lung glycerophospholipid species in mice exposed to hyperoxia as neonates**

All intact lipid species of phosphatidic acid (PA), phosphatidylcholine (PC), phosphatidylethanolamine (PE), phosphatidylglycerol (PG), phosphatidylinositol (PI) and phosphatidylserine (PS) from Table S1 are displayed. Data in heatmap is z-scored and sorted by component 1 of the principal component analysis. Each row represents the normalized intensities of a unique chromatographic feature. The features are color coded by row with green indicating high intensity, red indicating low intensity, and black indicating below limit of detection.

**
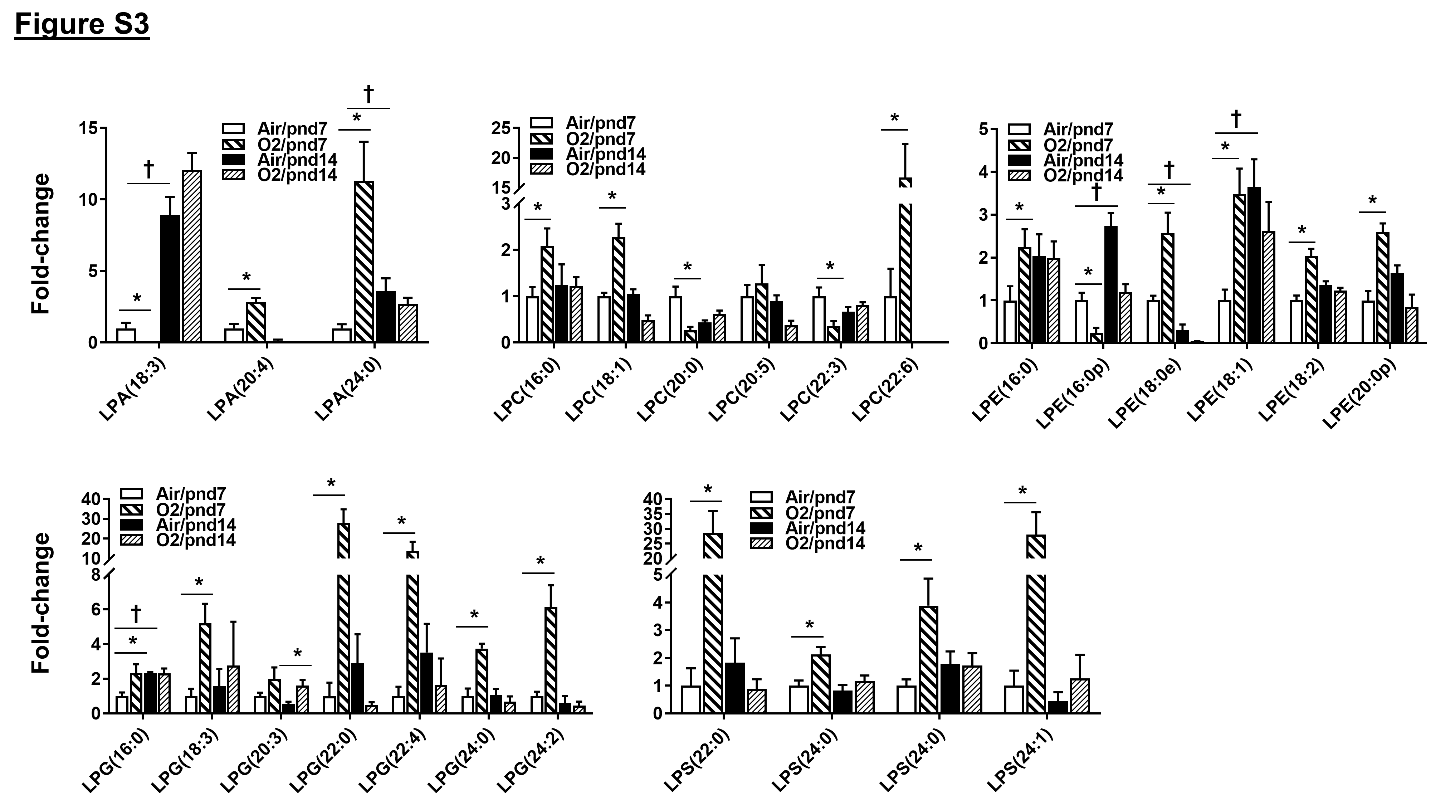
Figure S3. Compositional changes of lung glycerophospholipid species in mice exposed to hyperoxia as neonates**

LPA, LPC, LPE, LPG, and LPS species in mouse lungs with 2-fold change and *P*<0.05 between air and hyperoxia groups at pnd7 and pnd14 were listed. N=6. ^*^*P*<0.05 *vs* corresponding air; ^†^*P*<0.05 *vs* air/pnd7.

**
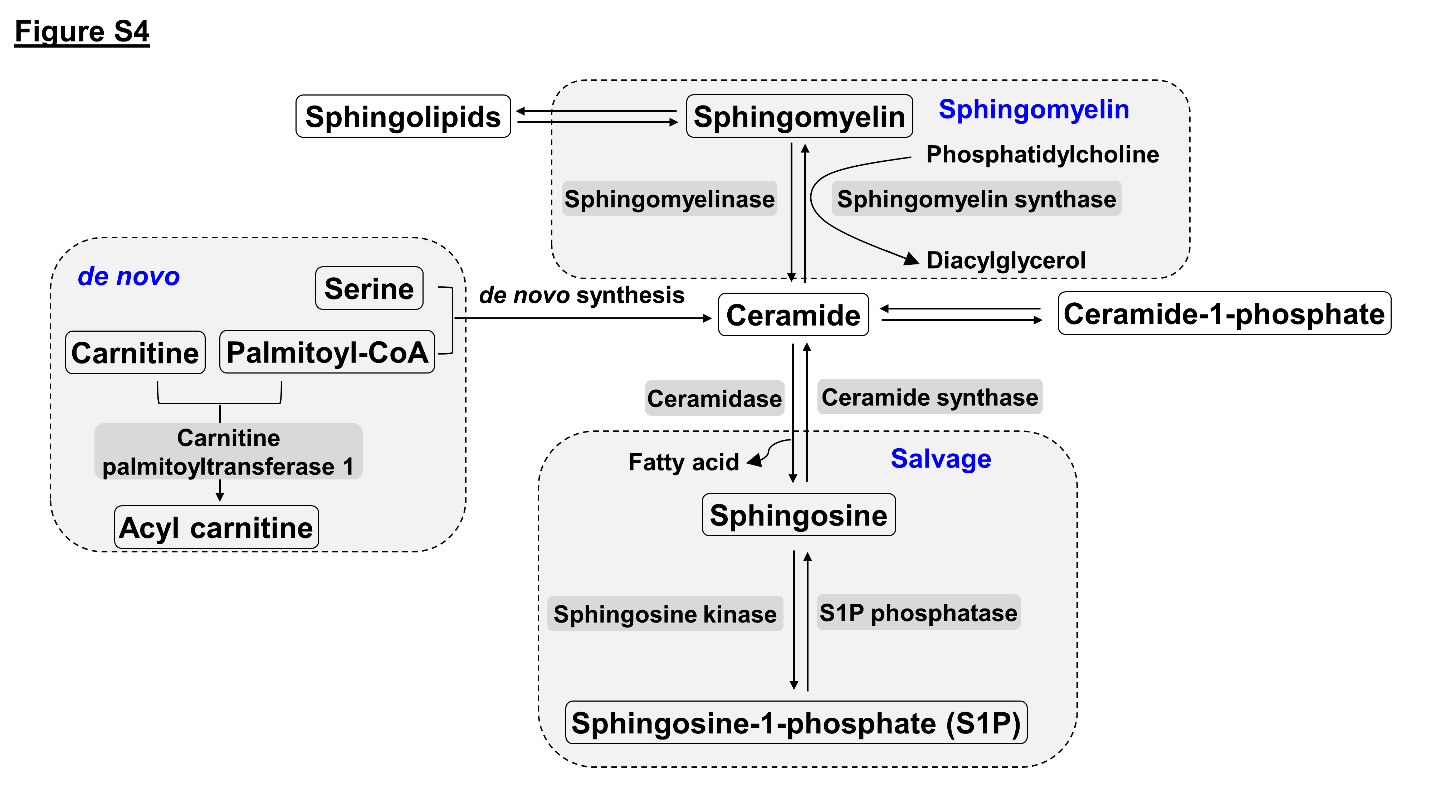
Figure S4. Sphingolipid metabolic pathway**

There are three major metabolic pathways involved in sphingolipid metabolism. This includes the de novo pathway coming from saturated fatty acids, the salvage pathway and the sphingomyelin pathway, all of which converge in ceramides.

**
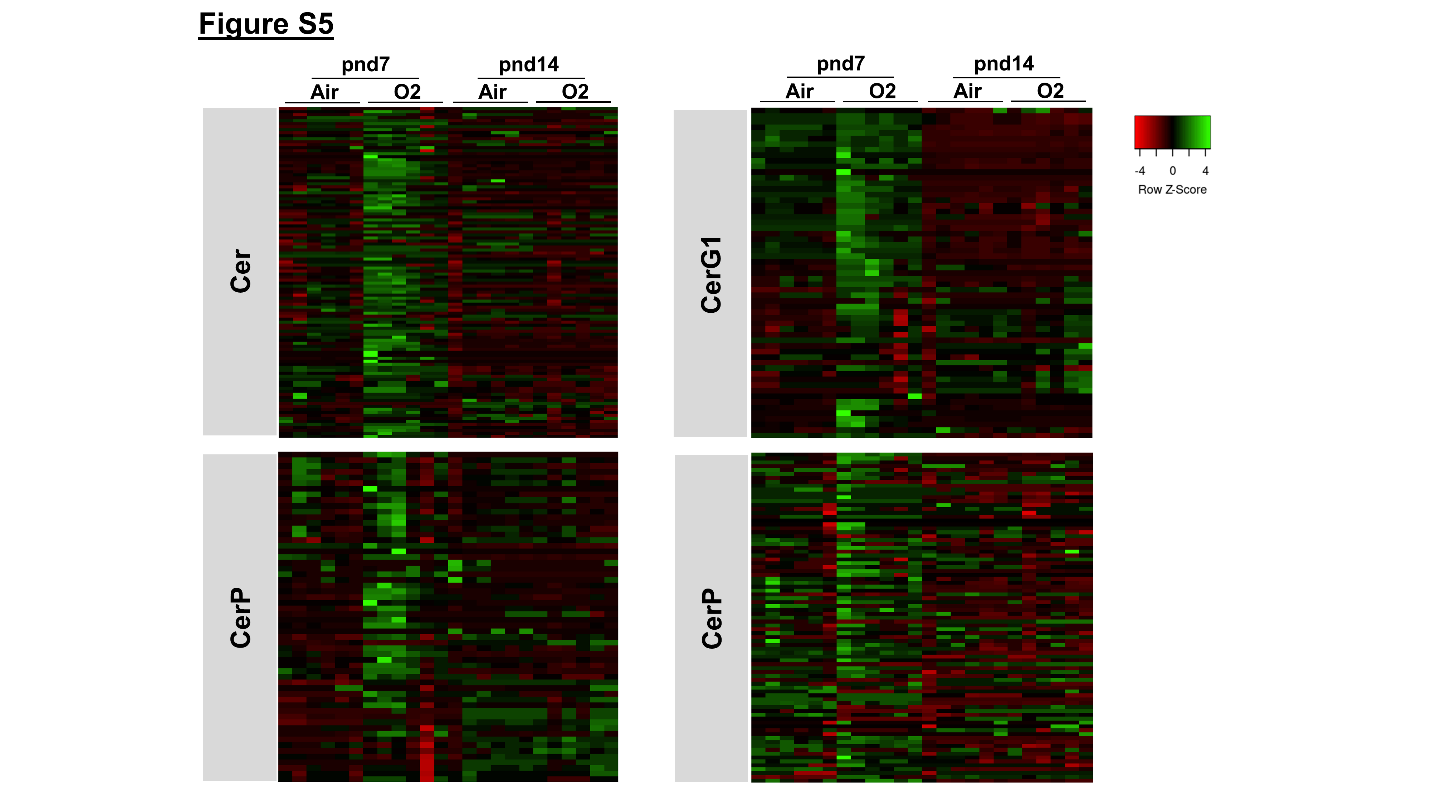
Figure S5. Lung sphingolipid species in mice exposed to hyperoxia as neonates**

All intact lipid species of ceramide (Cer), glucosylceramide (CerG1), ceramide phosphate (CerP), and sphingomyelin (SM) from Table S1 are displayed. Data in heatmap is z-scored and sorted by component 1 of the principal component analysis. Each row represents the normalized intensities of a unique chromatographic feature. The features are color coded by row with green indicating high intensity, red indicating low intensity, and black indicating below limit of detection.


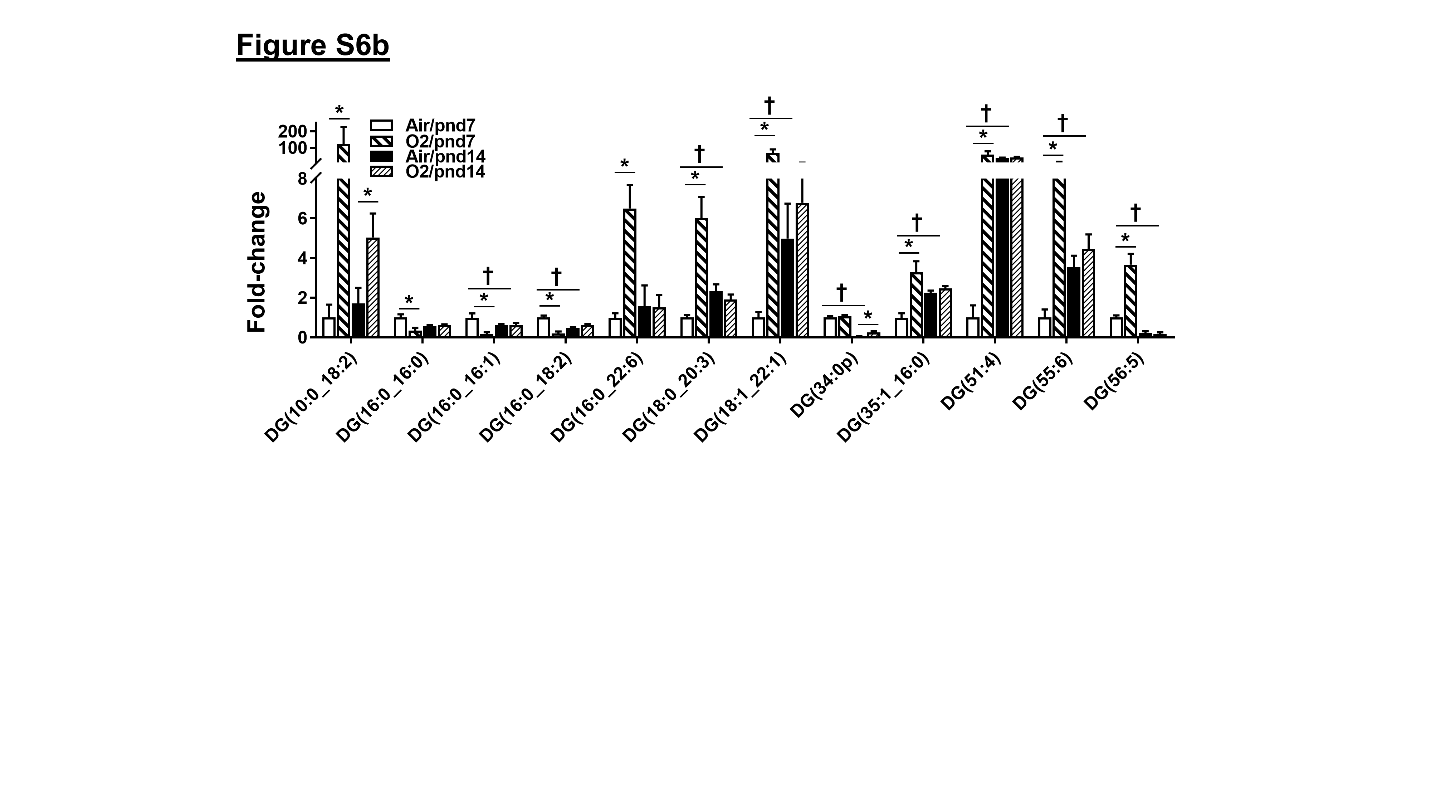

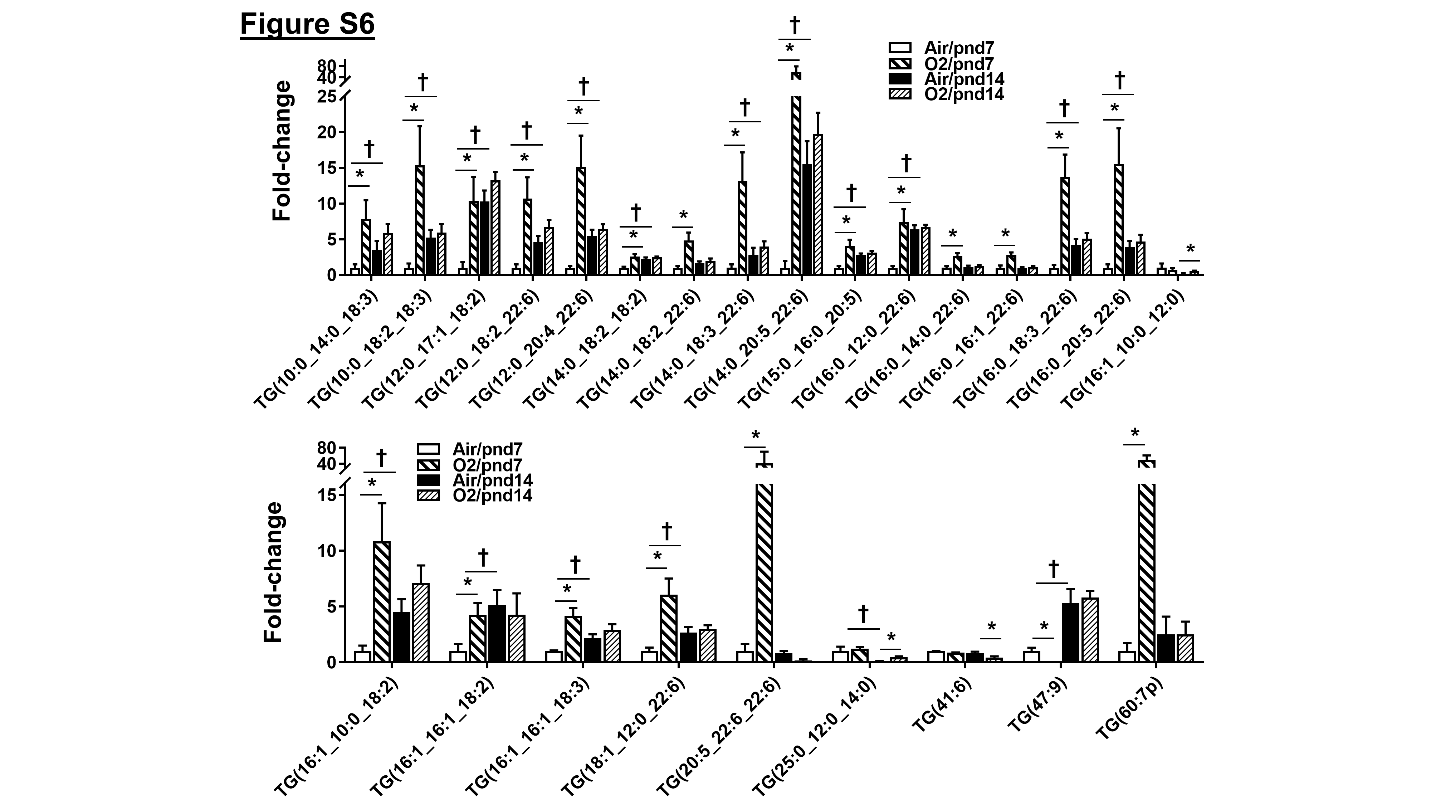
**Figure S6. Compositional changes of lung TG and DG species in mice exposed to hyperoxia as neonates**

TG and DG species in mouse lungs with 2-fold change and *P*<0.05 between air and hyperoxia groups at pnd7 and pnd14 were listed. N=6. ^*^*P*<0.05 *vs* corresponding air; ^†^*P*<0.05 *vs* air/pnd7.
